# Meta‐analysis of goal‐directed fluid therapy using transoesophageal Doppler monitoring in patients undergoing elective colorectal surgery

**DOI:** 10.1002/bjs5.50188

**Published:** 2019-07-04

**Authors:** K. E. Rollins, N. C. Mathias, D. N. Lobo

**Affiliations:** ^1^ Gastrointestinal Surgery, Nottingham Digestive Diseases Centre, National Institute for Health Research Nottingham Biomedical Research Centre Nottingham University Hospitals and University of Nottingham, Queen's Medical Centre Nottingham UK; ^2^ Medical Research Council/Arthritis Research UK Centre for Musculoskeletal Ageing Research, School of Life Sciences University of Nottingham, Queen's Medical Centre Nottingham UK; ^3^ University of Exeter Medical School Exeter UK

## Abstract

**Background:**

Intraoperative goal‐directed fluid therapy (GDFT) is recommended in most perioperative guidelines for intraoperative fluid management in patients undergoing elective colorectal surgery. However, the evidence in elective colorectal surgery alone is not well established. The aim of this meta‐analysis was to compare the effects of GDFT with those of conventional fluid therapy on outcomes after elective colorectal surgery.

**Methods:**

A meta‐analysis of RCTs examining the role of transoesophageal Doppler‐guided GDFT with conventional fluid therapy in adult patients undergoing elective colorectal surgery was performed in accordance with PRISMA methodology. The primary outcome measure was overall morbidity, and secondary outcome measures were length of hospital stay, time to return of gastrointestinal function, 30‐day mortality, acute kidney injury, and surgical‐site infection and anastomotic leak rates.

**Results:**

A total of 11 studies were included with a total of 1113 patients (556 GDFT, 557 conventional fluid therapy). There was no significant difference in any clinical outcome measure studied between GDFT and conventional fluid therapy, including overall morbidity (risk ratio (RR) 0·90, 95 per cent c.i. 0·75 to 1·08, *P* = 0·27; *I* ^2^ = 47 per cent; 991 patients), 30‐day mortality (RR 0·67, 0·23 to 1·92, *P* = 0·45; *I* ^2^ = 0 per cent; 1039 patients) and length of hospital stay (mean difference 0·01 (95 per cent c.i. −0·92 to 0·94) days, *P* = 0·98; *I* ^2^ = 34 per cent; 1049 patients).

**Conclusion:**

This meta‐analysis does not support the perceived benefits of GDFT guided by transoesophageal Doppler monitoring in the setting of elective colorectal surgery.

## Introduction

Intraoperative goal‐directed fluid therapy (GDFT) using measurements of stroke volume and cardiac output to inform administration of a small volume of fluid (usually 200–250 ml of a colloid, sometimes a crystalloid) to optimize stroke volume has been used for over two decades. A meta‐analysis^1^ published in 2014 of 22 RCTs that employed GDFT in a variety of elective and emergency operations showed that the risk of developing complications after surgery was reduced by 23 per cent when compared with conventional intraoperative fluid therapy. However, a further meta‐analysis^2^, which examined 23 RCTs including 2099 patients undergoing elective major abdominal surgery, suggested that GDFT may not be of benefit in all patients, particularly those managed in an enhanced recovery after surgery (ERAS)^3^, ^4^ setting. A recent RCT^5^ including 450 patients at low to moderate risk undergoing major elective surgery demonstrated that oesophageal Doppler‐guided GDFT was associated with a significant reduction in overall complications as well as length of hospital stay.

Most guidelines for perioperative care in colorectal surgery^3^, ^4^, ^6^, ^7^ recommend that GDFT should be used for patients undergoing colorectal surgery. Furthermore, the UK National Institute for Health and Care Excellence (NICE) has previously issued guidelines stating that intraoperative GDFT with transoesophageal Doppler monitoring should be used in ‘patients undergoing major or high‐risk surgery’^8^, with evidence supporting both a clinical benefit and cost‐saving based on the literature available when the guideline was published in 2011.

Previous meta‐analyses have been weakened by the inclusion of patients undergoing both emergency and elective surgery^1^, ^9^ and a wide range of operations^1^, ^2^, ^10^, and also by the fact that overall standards of perioperative care have changed over the past two decades. Moreover, the device used for monitoring stroke volume and cardiac output varied across the individual RCTs^2^. Hydroxyethyl starch (HES) was the colloid used frequently in the GDFT arm, but recently there has been a call by the European Medicines Agency for use of this fluid to be suspended^11^ because of the increased incidence of acute kidney injury (AKI) and the need for renal replacement therapy^12^, ^13^, ^14^.

The aim of this meta‐analysis of RCTs was to examine the effect of GDFT using transoesophageal Doppler monitoring compared with that of conventional intraoperative fluid therapy on postoperative outcome, including AKI, in patients undergoing elective major colorectal surgery.

## Methods

The protocol for this meta‐analysis was registered at the outset with the PROSPERO database (www.crd.york.ac.uk/prospero) (registration number CRD42018106818).

### Search strategy

A search of the PubMed, MEDLINE, Google™ Scholar and Cochrane Library databases was performed to identify full‐text studies evaluating the impact of intraoperative GDFT on postoperative surgical outcomes in patients undergoing elective colorectal surgery, published between January 1995 and July 2018. The electronic search terms used were [‘goal‐directed fluid therapy’ OR ‘flow‐directed fluid therapy’] AND [‘surgery’ OR ‘intraoperative’] AND [colon OR rectal OR colorectal]. No language restriction was imposed on the search. Only studies including adult patients undergoing elective colorectal surgery were selected. The bibliographies of all studies that met the inclusion criteria were hand‐searched for any additional suitable articles and relevant conference abstracts to ensure study inclusion was as complete as possible. The meta‐analysis was conducted according to the PRISMA statement^15^.

### Selection of articles

Following the exclusion of initial studies on the basis of article title and abstract by two independent researchers, the remaining full‐text articles were screened in detail for inclusion. Studies were included if they examined adult patients undergoing elective colorectal surgery who were randomized to receive either GDFT administered with transoesophageal Doppler monitoring or conventional intraoperative fluid therapy, and if the study reported at least one relevant postoperative outcome. Studies were excluded if any patient had undergone non‐colorectal surgery, or if they included any emergency surgical procedures, employed any device other than the transoesophageal Doppler for the conduct of GDFT, did not include any relevant clinical outcome measures, or if both groups received GDFT. Any studies in which inclusion criteria were not clear were discussed by the authors, with the final decision made by the senior author.

### Data extraction

Study data were extracted from the included RCTs by one author and checked by another. The primary outcome measure examined was overall postoperative morbidity; secondary outcome measures included 30‐day mortality, length of hospital stay (LOS), time to return of gastrointestinal function (flatus and stool), incidence of paralytic ileus and AKI, and rates of surgical‐site infection and anastomotic leak. Data were also collated on patient demographics (age, sex, ASA grade), surgical variables (surgical procedure, number of laparoscopic procedures, estimated blood loss) and intraoperative fluid administration (overall, maintenance and bolus fluid volumes, and inotrope administration). Data were extracted on whether the patient was managed using ERAS principles^16^, ^17^ or traditional perioperative care, and the method of administration of GDFT was noted. If data necessary for the conduct of the meta‐analysis were not available from the manuscript, the corresponding author was approached to obtain this with the aim of ensuring data collection was as complete as possible. If continuous data were reported only as median (i.q.r.) values and authors did not provide mean(s.d.) values, the technique described by Hozo *et al*.^18^ was used to estimate mean(s.d.) from median (i.q.r.) values. This technique uses the median as the best estimate of the mean, with the standard deviation calculated by the following formula: (upper limit of i.q.r. − lower limit of i.q.r.)/1·35.

Risk of bias was assessed using the Cochrane Collaboration tool for assessing bias from Review Manager version 5.3 (RevMan; The Cochrane Collaboration, The Nordic Cochrane Centre, Copenhagen, Denmark).

### Statistical analysis

Data extracted from the included studies were entered into the RevMan 5.3 software program. Dichotomous variables were analysed using the Mantel–Haenszel random‐effects model and quoted as a risk ratio (RR) with 95 per cent confidence intervals. Continuous variables were analysed using the inverse‐variance random‐effects model and quoted as a weighted mean difference (MD) with 95 per cent confidence intervals. When the Hozo technique^18^ had been used to estimate mean(s.d.) data from studies included to inform continuous data analysis, two separate meta‐analyses were conducted, one including the estimated data and one excluding these data. Data were used to construct forest plots, with *P* < 0·050 on two‐tailed testing indicating a statistically significant difference. Study heterogeneity and inconsistency were assessed using the *I* ^2^ statistic^19^, with 25 per cent or less representing low, 25–50 per cent moderate and above 50 per cent high heterogeneity.

## Results

**Figure 1:**
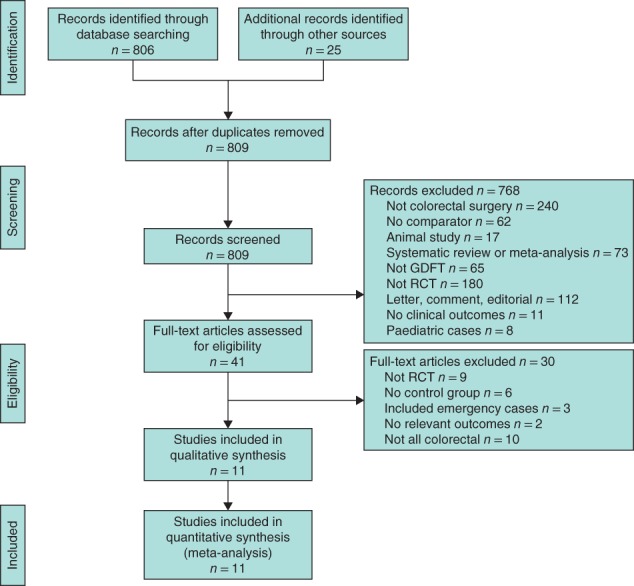
PRISMA diagram for the study GDFT, goal‐directed fluid therapy.

Of 831 studies identified initially, 11^20^, ^21^, ^22^, ^23^, ^24^, ^25^, ^26^, ^27^, ^28^, ^29^, ^30^ were deemed eligible for inclusion (*Fig*. 1). One published abstract^31^ was identified that would have been suitable for inclusion, but the data available from the text were insufficient to include in the meta‐analysis. Overall, the risk of bias of the studies included was low and generally the quality of the studies was medium to high (*Table* 1).

**Table 1:** Risk of bias in the 11 included studies

	Random sequence generation (selection bias)	Allocation concealment (selection bias)	Blinding of participants and personnel (performance bias)	Blinding of outcome assessment (detection bias)	Blinding of outcome data (attrition bias)	Selective reporting (reporting bias)	Other bias
Brandstrup *et al*.^23^	+	+	?	+	+	+	+
Challand *et al*.^25^	?	?	?	+	+	+	+
Conway *et al*.^30^	?	?	?	?	+	+	−
Gómez‐Izquierdo *et al*.^26^	+	+	+	+	+	+	+
Noblett *et al*.^28^	?	?	+	+	+	+	−
Phan *et al*.^21^	+	+	−	+	+	+	+
Reisinger *et al*.^20^	+	+	−	+	+	+	?
Senagore *et al*.^27^	+	?	?	?	+	+	+
Srinivasa *et al*.^22^	+	+	−	+	+	+	+
Wakeling *et al*.^29^	+	+	+	?	+	+	+
Zakhaleva *et al*.^24^	+	+	?	?	+	+	?

+, Low risk of bias; ?, uncertain risk of bias; −, high risk of bias.

### Demographics

The 11 RCTs in this meta‐analysis^20^, ^21^, ^22^, ^23^, ^24^, ^25^, ^26^, ^27^, ^28^, ^29^, ^30^ included a total of 1113 adult patients who had undergone elective colorectal surgery, of whom 556 were randomized to intraoperative GDFT using transoesophageal Doppler monitoring and 557 to traditional intraoperative fluid management strategies. In ten studies GDFT was administered as part of an enhanced recovery programme, with just one study^30^ being conducted within a traditional care programme; thus no analysis was conducted comparing patients managed as part of these differing pathways. Data on laparoscopic surgical approach only were provided by two studies^26^, ^27^, with no studies providing data from open‐only approaches. Baseline patient demographics are provided in *Table* S1 (supporting information), and fluid administered during the perioperative period is detailed in *Table* S2 (supporting information).

### Overall morbidity

**Figure 2:**
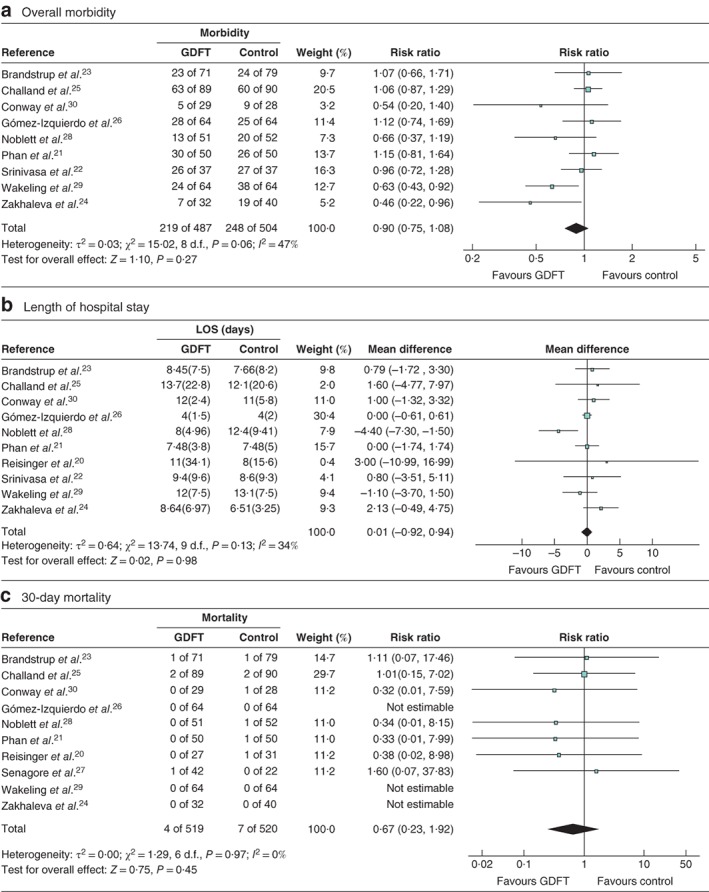
Forest plots of overall morbidity, length of hospital stay and 30‐day mortality **a** Overall morbidity, **b** mean(s.d.) length of hospital stay (LOS) and **c** 30‐day mortality in patients receiving goal‐directed fluid therapy (GDFT) *versus* controls. **a,c** Risk ratios are shown with 95 per cent confidence intervals; a Mantel–Haenszel random‐effects model was used for meta‐analysis. **b** Mean differences are shown with 95 per cent confidence intervals; an inverse‐variance random‐effects model was used to perform the meta‐analysis.

Nine studies^21^, ^22^, ^23^, ^24^, ^25^, ^26^, ^28^, ^29^, ^30^ including 487 patients managed with GDFT and 504 who had traditional fluid management reported overall morbidity rates (*Fig*. 2 *a*). Overall morbidity was not significantly different between these groups (RR 0·90, 95 per cent 0·75 to 1·08, *P* = 0·27; *I* ^2^ = 47 per cent).

### Length of hospital stay

Overall LOS was considered by ten studies^20^, ^21^, ^22^, ^23^, ^24^, ^25^, ^26^, ^28^, ^29^, ^30^ included in the meta‐analysis (1049 patients, 514 GDFT and 535 traditional) (*Fig*. 2 *b*). However, two studies^20^, ^26^ included only median (i.q.r.) data and did not provide the authors with mean(s.d.) data for the meta‐analysis. These data were estimated using the technique described by Hozo *et al*.^18^ and all data were included in the primary analysis of LOS. A separate meta‐analysis was performed for this outcome excluding the estimated data.

In the first analysis, which included estimated data for LOS, GDFT was not associated with a significant difference in the overall group (MD 0·01 (95 per cent c.i. −0·92 to 0·94) days, *P* = 0·98; *I* ^2^ = 34 per cent) (*Fig*. 2 *b*). In the second analysis, which excluded the estimated data, GDFT resulted in no significant change in hospital length of stay (MD −0·01 (−1·38 to 1·35) days, *P* = 0·99; *I* ^2^ = 48 per cent).

### Thirty‐day mortality

Mortality rates were detailed in ten studies^20^, ^21^, ^23^, ^24^, ^25^, ^26^, ^27^, ^28^, ^29^, ^30^, including 519 patients in the GDFT group and 520 in the traditional group (*Fig*. 2 *c*). Overall there was no significant difference in 30‐day mortality between GDFT and control patients (RR 0·67, 95 per cent c.i. 0·23 to 1·92, *P* = 0·45; *I* ^2^ = 0 per cent).

### Surgical‐site infection

**Figure 3:**
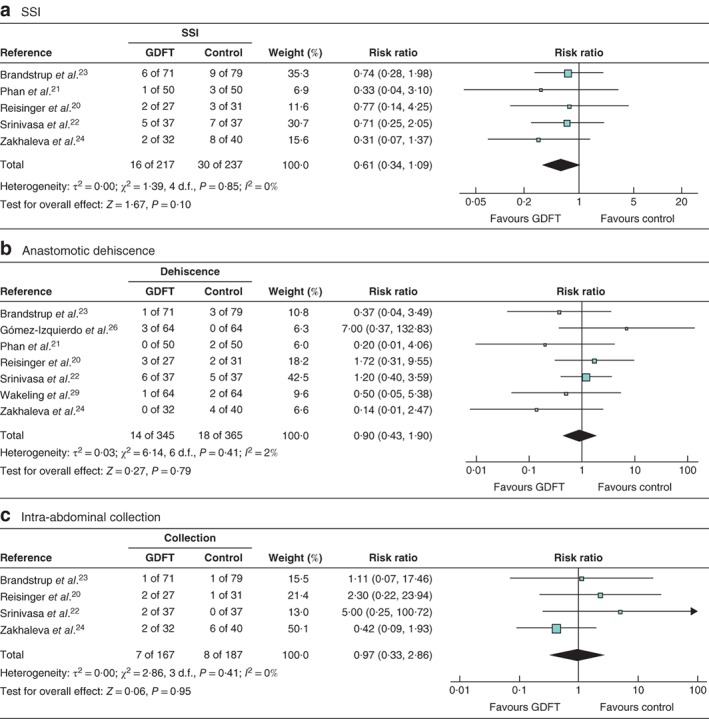
Forest plots of surgical‐site infection, anastomotic dehiscence and intra‐abdominal collection **a** Surgical‐site infection (SSI), **b** anastomotic dehiscence and **c** intra‐abdominal collection in patients receiving goal‐directed fluid therapy (GDFT) *versus* controls. Risk ratios are shown with 95 per cent confidence intervals. Mantel–Haenszel random‐effects models were used for meta‐analysis.

Five studies^20^, ^21^, ^22^, ^23^, ^24^ examined the incidence of surgical‐site infection: 217 patients managed with intraoperative GDFT *versus* 237 controls (*Fig*. 3 *a*). Use of GDFT did not affect the rate of surgical‐site infection significantly (RR 0·61, 95 per cent c.i. 0·34 to 1·09, *P* = 0·10; *I* ^2^ = 0 per cent).

### Anastomotic dehiscence

Seven studies^20^, ^21^, ^22^, ^23^, ^24^, ^26^, ^29^ included data on the rate of anastomotic dehiscence: 345 patients managed with intraoperative GDFT *versus* 365 control patients (*Fig*. 3 *b*). Intraoperative administration of GDFT did not affect the incidence of anastomotic dehiscence (RR 0·90, 95 per cent c.i. 0·43 to 1·90, *P* = 0·79; *I* ^2^ = 2 per cent).

### Intra‐abdominal collection

A total of four studies^20^, ^22^, ^23^, ^24^ examined the relationship between GDFT and conventional fluid therapy and the rate of intra‐abdominal collection (*Fig*. 3 *c*). There was no significant difference between the two groups (RR 0·97, 95 per cent c.i. 0·33 to 2·86, *P* = 0·95; *I* ^2^ = 0 per cent).

### Postoperative ileus

**Figure 4:**
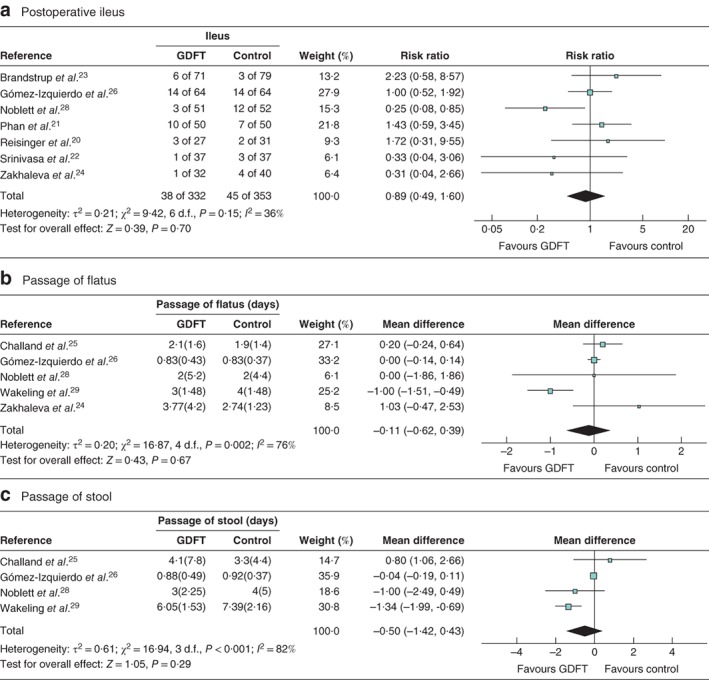
Forest plots of postoperative ileus, time to passage of flatus and time to passage of stool **a** Postoperative ileus, **b** mean(s.d.) time to passage of flatus and **c** mean(s.d.) time to passage of stool in patients receiving goal‐directed fluid therapy (GDFT) *versus* controls. **a** Risk ratios are shown with 95 per cent confidence intervals; a Mantel–Haenszel random‐effects model was used for meta‐analysis. **b,c** Mean differences are shown with 95 per cent confidence intervals; inverse‐variance random‐effects models were used to perform the meta‐analyses.

Seven studies^20^, ^21^, ^22^, ^23^, ^24^, ^26^, ^28^ included data on the rate of postoperative ileus: 332 patients managed with intraoperative GDFT *versus* 353 control patients (*Fig*. 4 *a*). The use of GDFT did not affect the incidence of postoperative paralytic ileus significantly (RR 0·89, 95 per cent c.i. 0·49 to 1·60, *P* = 0·70; *I* ^2^ = 36 per cent).

### Return of gastrointestinal function

Five studies examined time to return of gastrointestinal function after surgery, in either the form of flatus^24^ or both flatus and stool^25^, ^26^, ^28^, ^29^. Two separate analyses were performed for each outcome as in previous analyses.

For time to flatus in all studies, including those with calculated data (*Fig*. 4 *b*), there were 300 patients managed with GDFT and 310 control patients in five studies^24^, ^25^, ^26^, ^28^, ^29^. There was no significant difference in the time to return of flatus between the two fluid strategies (MD −0·11 (95 per cent c.i. −0·62 to 0·39) days, *P* = 0·67; *I* ^2^ = 76 per cent). Just two studies^25^, ^29^ were left when data calculated using the Hozo technique^18^ were excluded, so a further meta‐analysis was not attempted.

When time to stool was considered, 268 patients were managed with GDFT and 270 with control intraoperative fluid (*Fig*. 4 *c*). There was no significant difference in the overall group (MD −0·50 (95 per cent c.i. −1·42 to 0·43) days, *P* = 0·29; *I* ^2^ = 82 per cent). When data for the single study^26^ with estimated data were excluded, no difference in time to passage of stool was observed (MD −0·76 (−1·88 to 0·35) days, *P* = 0·18; *I* ^2^ = 56 per cent).

### Acute kidney injury

Four studies^21^, ^24^, ^25^, ^29^ examined the relationship between GDFT and conventional fluid therapy and the incidence of AKI in 479 patients; there was no significant difference between the two groups (RR 1·51, 95 per cent c.i. 0·85 to 2·66, *P* = 0·16; *I* ^2^ = 0 per cent).

## Discussion

This meta‐analysis has demonstrated that in patients undergoing elective colorectal surgery GDFT guided by transoesophageal Doppler monitoring was not associated with a significant difference in any postoperative clinical outcome measure compared with conventional intraoperative fluid therapy. There was an indication of difference in terms of a reduced rate of surgical‐site infection when patients received GDFT, and increased rate of AKI, but neither of these trends was statistically significant. Data on the role of GDFT in the open *versus* the laparoscopic approach for elective colorectal surgery were insufficient, so the decision was taken not to perform a meta‐analysis for this variable. Further high‐quality evidence is necessary to assess the potential role of GDFT between surgical approaches. Only one study^30^ examined the role of GDFT as part of a traditional care pathway *versus* ten studies within an ERAS pathway; hence no comparison could be made between these settings.

These findings are more pronounced in the lack of benefit for GDFT than found in a previous meta‐analysis^2^ examining the role of GDFT in elective abdominal surgery, although that study^2^ did not limit the papers included by the method of administration of GDFT. The previous meta‐analysis^2^ found that, overall, GDFT was associated with a significant reduction in morbidity, LOS, duration of stay in the ICU and time to passage of stool, with no significant difference observed in the incidence of postoperative ileus, mortality or time to return of flatus.

An issue raised by other meta‐analyses^2^, ^9^, ^10^ regarding the comparison between GDFT and traditional fluid management strategies is differences in fluid management strategies over the past three decades. In the more historical studies, large volumes tended to be infused in the traditionally managed group, whereas more contemporary fluid management strategies observed in more recently published studies tend to aim for zero balance and for the patient to reach the anaesthetic room in a well hydrated state. The U‐shaped relationship between fluid volume infused and perioperative morbidity that has been postulated previously^32^ may suggest that in more modern fluid management strategies^33^, ^34^ the differences in clinical benefit between GDFT and traditional intraoperative fluid management may not be as pronounced if fluid overload and deficits are avoided.

This study is the largest to examine the impact of GDFT *versus* traditional fluid management strategies in elective colorectal surgery. The only other previous meta‐analysis^35^ published on the topic has several methodological issues, as it inadvertently included studies that were not RCTs, despite this being the stated aim, and missed several key studies on the topic. The present study included just one method for the conduct of GDFT: transoesophageal Doppler monitoring. This is the commonest device used to perform GDFT in the UK, as well as in widespread use internationally^36^, and is the method suggested by NICE in the UK^8^. Evidence has suggested that transoesophageal Doppler and other methods for GDFT such as lithium dilution techniques^37^, ^38^, calibrated pulse contour analysis^39^ and the pleth variability index^40^ are not interchangeable; hence the decision was taken to make the study population as homogeneous as possible.

Generally, the degree of heterogeneity within the analyses conducted was low, with five analyses having low levels of heterogeneity, four having moderate levels, and just three having high levels of heterogeneity (those conducted on time to passage of flatus and stool). This large degree of concordance in study results, in addition to the relatively high study quality, adds weight to the conclusions drawn.

This meta‐analysis has a few weaknesses inherent in its design and conduct. The regimens for postoperative fluid management were poorly documented in the included studies. This may have impacted on postoperative outcome, but cannot be accounted for. This study chose to focus on just one technique for the conduct of GDFT, transoesophageal Doppler monitoring, as this was felt to improve the homogeneity of the meta‐analysis. However, this restricts the generalizability of the results to GDFT conducted using other techniques. In addition, efforts to obtain raw data for the continuous variables were not successful. Therefore, the Hozo technique^18^ was used to estimate mean(s.d.) data for two studies^20^, ^26^ in the LOS analysis, three studies^26^, ^28^, ^29^ in the time to return of flatus, and one study^26^ in the time to return of stool. To negate this potential weakness, an additional analysis, excluding the estimated data, was planned at the outset of the conduct of the meta‐analysis. For the LOS analysis and time to return of stool, excluding the estimated data, no difference was observed in the outcome. However, as three of the five studies required estimated data in the time to return of flatus analysis, no further analysis was conducted due to the poor strength of any conclusion drawn.

A similar meta‐analysis^35^, including 11 RCTs, was published in 2018 examining the role of GDFT in colorectal surgery. However, two of these 11 studies were not RCTs: one^41^ was a comparison between a series of patients recruited to undergo GDFT and a historical series of patients managed with traditional fluid management, and the other^42^ was a matched cohort of patients who were not randomized. In addition, three papers^20^, ^21^, ^22^, which should have been included as they met the inclusion criteria, were missed. They included a study^30^ of patients undergoing ‘major bowel surgery’, which was excluded from the present meta‐analysis. All but one study used transoesophageal Doppler‐guided GDFT, with the single remaining study^43^ including central venous oxygen saturation‐guided GDFT; however, this was excluded from the present meta‐analysis. The above‐mentioned methodological flaws in this paper^35^ weaken the strength of the conclusions.

The nature of the fluid used for the bolus associated GDFT was variable, with HES being the documented fluid administered in seven studies^20^, ^21^, ^23^, ^25^, ^26^, ^27^, ^30^. However, there is a moratorium on the use of HES owing to concerns of an increased risk of AKI requiring renal replacement therapy^12^, ^13^, ^14^ as well as mortality^12^, ^14^, based on recent RCTs. The indication towards increasing rates of AKI in patients receiving GDFT compared with those having traditional fluid management could potentially be related to the larger volume of HES infused in this group and the inherent increased risks. Just four studies^21^, ^24^, ^25^, ^29^ included data on AKI rates, with the nature of the bolus fluid being variably reported in these. In one study^25^ HES was administered for boluses, one^21^ gave a colloid although the choice was ‘at the discretion of the anaesthetist’ and included HES, gelatine or human albumin solution, one^29^ gave non‐HES colloid, and one^24^ did not specify the type of colloid given. If just those two studies^21^, ^25^ clearly receiving HES were analysed for AKI, the strength of the indication increased; however, based on just two studies, this is far from conclusive evidence. Future studies focusing on the role of GDFT will not include HES as the bolus agent^11^, and this may result in improved clinical outcomes.

This study has demonstrated no benefit for the routine use of transoesophageal Doppler‐guided GDFT in patients undergoing elective colorectal surgery, contrary to NICE guidance^8^, which recommends that GDFT technology should be used ‘in patients undergoing major or high‐risk surgery’.

## Supporting information


**Table S1** Baseline patient demographics for all included studies
**Table S2** Intraoperative fluid infused in goal‐directed and control groups
