# Supplementary material for: Meta‐analysis of goal‐directed fluid therapy using transoesophageal Doppler monitoring in patients undergoing elective colorectal surgery
Source: BJS Open. 2019 Jul 4;3(5):606–16. doi: 10.1002/bjs5.50188 (PMC6773648; doi:10.1002/bjs5.50188)
Supplement: Supplementary file 1 — Table S1 Baseline patient demographics for all included studies Table S2 Intraoperative fluid infused in goal‐directed and control groups [file BJS5-3-606-s001.docx]

BJS5_50188

**Meta-analysis of goal-directed fluid therapy using transoesophageal Doppler monitoring in patients undergoing elective colorectal surgery**

**K. E. Rollins, N. C. Mathias and D. N. Lobo**

**Table S1 Baseline patient demographics for all included studies**

|  | Perioperative Care Pathway | Number of Patients | | Type of Surgery | | Laparoscopic Approach | | ASA 1:2:3:4 | |
| --- | --- | --- | --- | --- | --- | --- | --- | --- | --- |
| Reference | ERAS or Traditional | GDFT | Control | GDFT | Control | GDFT | Control | GDFT | Control |
| Brandstrup *et al.* 2012^23^ | ERAS | 71 | 79 | All elective colorectal | All elective colorectal | 32, additional 11 converted from laparoscopic to open | 38, additional 12 converted from laparoscopic to open | 26:37:8:0 | 20:43:16:0 |
| Challand *et al.* 2012^25^ | ERAS | 89 | 90 | 32 colonic, 57 rectal (65 carcinoma) | 37 colonic, 53 rectal (68 carcinoma) | 28 | 37 | 11:51:27 (3+4) | 11:52:27 (3+4) |
| Conway *et al.* 2002^30^ | Traditional | 29 | 28 | All major bowel surgery | All major bowel surgery | Not stated | Not stated | Median (range) ASA class 1 (1-3) | Median (range) ASA class 2 (1-3) |
| Gomez-Izquierdo *et al.* 2017^26^ | ERAS | 64 | 64 | 39 colonic resection, 25 rectal resection | 39 colonic resection, 25 rectal resection | 64 laparoscopic, of which 8 converted to open | 64 laparoscopic, of which 5 converted to open | 6:42:14:2 | 8:38:18:0 |
| Noblett *et al.* 2006^28^ | ERAS | 51 | 52 | 30 colonic, 24 rectal | 25 colonic, 29 rectal | 13 | 13 | Mean (SD) ASA class 2.1 (0.6) | Mean (SD) ASA class 2.2 (0.6) |
| Phan *et al.* 2014^21^ | ERAS | 50 | 50 | 12 right hemicolectomy, 17 anterior resection, 21 other (29 cancer surgery) | 14 right hemicolectomy, 22 anterior resection, 1 abdominal/perineal, 13 other (34 cancer surgery) | 31 (additional 8 converted to open) | 28 (additional 8 converted to open) | Median (range) ASA class 2 (1-3) | Median (range) ASA class 2 (1-3) |
| Reisinger *et al.* 2017^20^ | ERAS | 27 | 31 | 10 right hemicolectomy, 1 left hemicolectomy, 5 sigmoid colectomy, 11 rectal resection | 12 right hemicolectomy, 0 left hemicolectomy, 5 sigmoid colectomy, 13 rectal resection, 1 subtotal colectomy | 7, of which 0 converted to open | 10, of which 4 converted to open | 8:14:5:0 | 4:24:3:0 |
| Senagore *et al.* 2009^27^ | ERAS | 42 | 22 | All colectomy | All colectomy | 42 | 42 | Not stated | Not stated |
| Srinivasa *et al.* 2013^22^ | ERAS | 37 | 37 | 14 right hemicolectomy, 4 extended right hemicolectomy, 14 high anterior resection, 5 total/subtotal colectomy | 17 right hemicolectomy, 5 extended right hemicolectomy, 14 high anterior resection, 1 total/subtotal colectomy | 5 | 6 | 5:20:12:0 | 5:15:17:0 |
| Wakeling *et al.* 2005^29^ | ERAS | 64 | 64 | 31 anterior and AP resection, 15 left hemicolectomy and sigmoid colectomy, 15 right hemicolectomy, 3 reversal of Hartmann’s | 33 anterior and AP resection, 15 left hemicolectomy and sigmoid colectomy, 9 right hemicolectomy, 4 subtotal colectomy, 2 reversal of Hartmann’s, 1 Crohn's resection | not stated | not stated | Median (IQR) ASA class 2 (1) | Median (IQR) ASA class 2 (1) |
| Zakhaleva *et al.* 2013^24^ | ERAS | 32 | 40 | 24 colectomy, 7 proctectomy, 1 small bowel resection - 16 malignant, 11 benign, 5 IBD | 30 colectomy, 6 proctectomy, 4 small bowel resection - 17 malignant, 19 benign, 4 IBD | 16 + 2 converted to open | 19 + 5 converted to open | 0:7:26:0 | 0:7:32:0 |

AP =abdomino perineal, ASA=American Society of Anesthesiologists, ERAS=Enhanced Recovery After Surgery, IBD=inflammatory bowel disease, IQR=interquartile range, SD=standard deviation

**Table S2 Intraoperative fluid infused in goal-directed and control groups**

|  | Total fluid volume infused (ml) | | Total crystalloid volume (ml) | | Total colloid bolus volume (ml) | | Blood loss (ml) | | Requirement for perioperative inotropes | |
| --- | --- | --- | --- | --- | --- | --- | --- | --- | --- | --- |
| Reference | GDFT | Control | GDFT | Control | GDFT | Control | GDFT | Control | GDFT | Control |
| Brandstrup *et al.* 2012^23^ | Mean 1876* | Mean 1491* | Saline and lactated Ringer’s: Mean (SD) 483 (419) | Saline and lactated Ringer’s: Mean (SD) 443 (480) | Mean (SD) 810 (543)* | Mean (SD) 475 (598)* | Not stated | Not stated | Not stated | Not stated |
| Challand *et al.* 2012^25^ | Not stated | Not stated | Mean (SD) 3479 (1181) | Mean (SD) 3593 (1398) | Mean (SD) 1718 (446)* | Mean (SD) 336 (623)* | Median (IQR) 500 (200-1000)* | Median (IQR) 250 (100-500)* | Not stated | Not stated |
| Conway *et al.* 2002^30^ | Mean (SD) 64.6 (36.4) ml/kg | Mean (SD) 55.2 (24) ml/kg | Not stated | Not stated | Mean (SD) 28 (16) ml/kg | Mean (SD) 19 (15) ml/kg | Not stated | Not stated | Not stated | Not stated |
| Gomez-Izquierdo *et al.* 2017^26^ | Median (IQR) 1535 (1000-2272)* | Median (IQR) 2370 (1779-3071)* | Median (IQR) Lactated Ringer’s: 500 (323-687)*  Saline: 194 (150-268) | Median (IQR) Lactated Ringer’s: 2102 (1600-2528)*  Saline: 179 (146-234) | Median (IQR) 900 (400-1400)* | Median (IQR) 0 (0-500)* | Median (IQR) 175 (100-400) | Median (IQR) 150 (100-400) | Phenylephrine n=53 (83%)  Ephedrine n=40 (62%) | Phenylephrine n=58 (91%)  Ephedrine n=43 (67%) |
| Noblett *et al.* 2006^28^ | Not stated | Not stated | Mean (SD) 2298 (863) | Mean (SD) 2625 (1004) | Mean (SD) 1340 (838) | Mean (SD) 1209 (824) | Median (IQR) 250 (40-2455) | Median (IQR) 475 (100-2900) | n=16 (31%)* | n=26 (50%)* |
| Phan *et al.* 2014^21^ | Median (IQR) 2190 (1350-2560)* | Median (IQR) 1500 (1200-2000) * | Median (IQR) 1500 (1000-2000) | Median (IQR) 1400 (1000-1900) | Median (IQR) 500 (250-750)* | Median (IQR) 0 (0-300)* | Not stated | Not stated | Not stated | Not stated |
| Reisinger *et al.* 2017^20^ | Mean (SD) 14. (4.7) ml/kg/h | Mean (SD) 16.2 (5.9) ml/kg/h | Mean (SD) 3000 (1093) | Mean (SD) 3026 (1307) | Mean (SD) 1526 (823) | Mean (SD) 952 (687) | Mean (SD) 957 (1880) | Mean (SD) 461 (1026) | n=12 (44.4%) | n=15 (48.4%) |
| Senagore *et al.* 2009^27^ | Not stated | Not stated | Not stated | Not stated | Not stated | Not stated | Not stated | Not stated | Not stated | Not stated |
| Srinivasa *et al.* 2013^22^ | Mean (SD) 1994 (590)* | Mean (SD) 1614 (420)* | Not stated | Not stated | Mean (SD) 591 (471)* | Mean (SD) 297 (275)* | Not stated | Not stated | n=31 (83.7%) | n=34 (91.9%) |
| Wakeling *et al.* 2005^29^ | Not stated | Not stated | Median 3000 | Median 3000 | Median 2000* | Median 1500* | Median (IQR) 500 (700) | Median (IQR) 500 (975) | Not stated | Not stated |
| Zakhaleva *et al.* 2013^24^ | Median (IQR) 3100 (700-77000) | Median (IQR) 4000 (900-6200) | Median (IQR) 2700 (500-6500) | Median (IQR) 3200 (500-5600) | Median (IQR) 500 (0-2800)* | Median (IQR) 300 (0-4500)* | Median (IQR) 100 (10-650) | Median (IQR) 100 (10-500) | Not stated | Not stated |

*Indicates statistically significant difference between GDFT and Control groups

IQR=interquartile range, SD=standard deviation
